# An Unusual Case of Prosthetic Valve Endocarditis

**DOI:** 10.7759/cureus.25735

**Published:** 2022-06-07

**Authors:** Noelle Provenzano, James Boris, Bharghava Nelluri, Lorin Berman, Raymond Singer, Hojoon You

**Affiliations:** 1 Internal Medicine, Einstein Medical Center Montgomery, East Norriton, USA; 2 Cardiothoracic Surgery, Einstein Medical Center Montgomery, East Norriton, USA; 3 Infectious Disease, Einstein Medical Center Montgomery, East Norriton, USA

**Keywords:** prosthetic valve infective endocarditis, mycobacterium chimaera, surgical complication, valve repair, endocarditis

## Abstract

*Mycobacterium chimaera *is a slow-growing nontuberculous mycobacterium. It has been identified as a contaminant during open-heart surgery. It contaminates water in heater-cooler units that then become aerosolized, contaminating the surgical field. We report a 56-year-old male who presented with culture-negative endocarditis six years after his initial open-heart surgery.

## Introduction

*Mycobacterium chimaera* is a slow-growing nontuberculous *Mycobacterium avium *complex. Evidence suggests that it is responsible for causing prosthetic valve endocarditis, kidney and liver dysfunction, osteomyelitis, and other life-threatening conditions. Since 2006, the transmission of *M.chimaera* during open-heart surgery has been recognized. A case series from 30 patients with *M.chimaera* detected in any clinical specimen, history of cardiothoracic surgery with cardiopulmonary bypass, and compatible clinical presentation in the United Kingdom found that 14 patients exhibited prosthetic valve endocarditis [1]. Between 2013 and 2017, more than 100 cases linked to open-heart surgery were identified. It has been hypothesized that the organism contaminated the water used in the heater-cooler units (HCUs) used during open-heart surgery. The organism likely became aerosolized through breaches in the HCU water tanks and contaminated the surgical field [2]. Although the infection’s incubation period has not been widely publicized, one case noted that the infective endocarditis did not become symptomatic until 12 years after the initial surgery [3]. We outline a patient who experienced culture-negative endocarditis six years after his initial surgery.

## Case presentation

A 56-year-old male with a past medical history significant for mitral valve repair followed by replacement, atrial fibrillation, and alcoholism, presented with shortness of breath in October 2020. He had significant cardiac and respiratory complications before this presentation. 

In 2014 he underwent his first mitral valve repair with an annuloplasty ring. In the years following, he subsequently suffered from multiple embolic events including strokes. A transesophageal echocardiogram at that time revealed the source of the emboli to be endocarditis of the valve with thrombus formation. He fully recovered from the strokes caused by the emboli. In August of 2019, he again presented with dyspnea. His echocardiogram demonstrated moderate mitral regurgitation along with severe mitral stenosis with a high gradient. The patient required a mitral valve replacement, however, he was reluctant to have a mechanical valve placed. The patient then underwent mitral valve replacement with a bovine bioprosthesis. During the procedure, it was found that the vegetation due to chronic infection, extended from the previous annuloplasty ring. The vegetations caused dissociation from the original mitral annulus and the free wall of the left ventricle; this required the posterior annulus to be reconstructed using a bovine pericardial patch. Work-up for the vegetations at the time was unrevealing, including 16S ribosomal amplification, *Bartonella* serologies, as well as fungal and acid-fast bacilli (AFB) cultures. The patient was discharged home in stable condition. 

**Figure 1:**
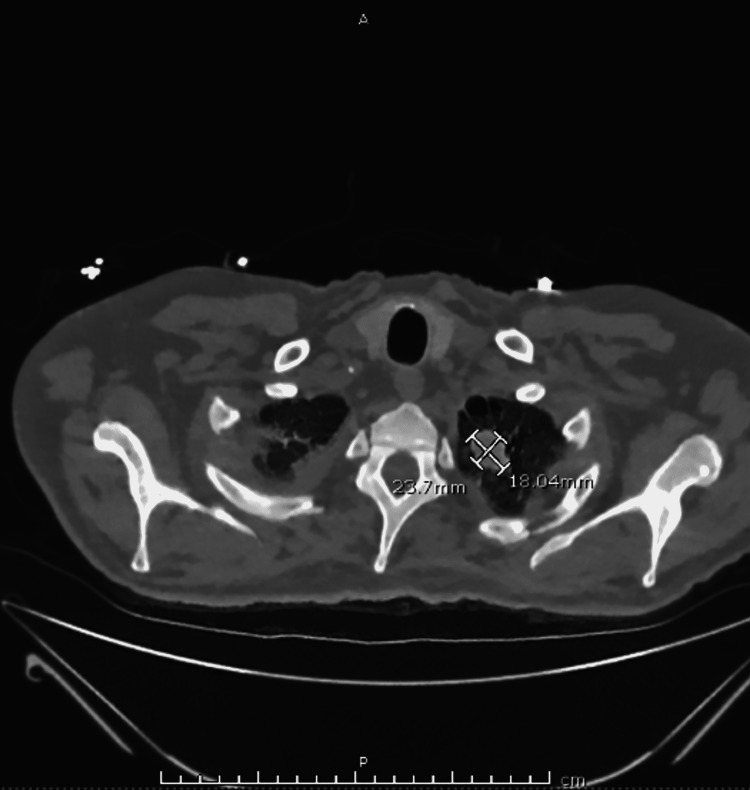
A computed tomography scan showing an increase in the size of a known left upper lobe nodule

In October of 2020, the patient was diagnosed with hemophagocytic lymphohistiocytosis and successfully completed a dexamethasone taper. Two weeks after this diagnosis, he presented to the emergency department for evaluation of generalized weakness. In the intermittent two-week period, he experienced extreme fatigue, dyspnea, and episodes of fever and chills accompanied by drenching sweats at home. Due to the patient's intermittent fevers, blood cultures were drawn. A computed tomography scan to further evaluate his dyspnea revealed an extensive pulmonary embolism, and an increase in the size of a known left upper lobe nodule (Figure 1).

**Figure 2:**
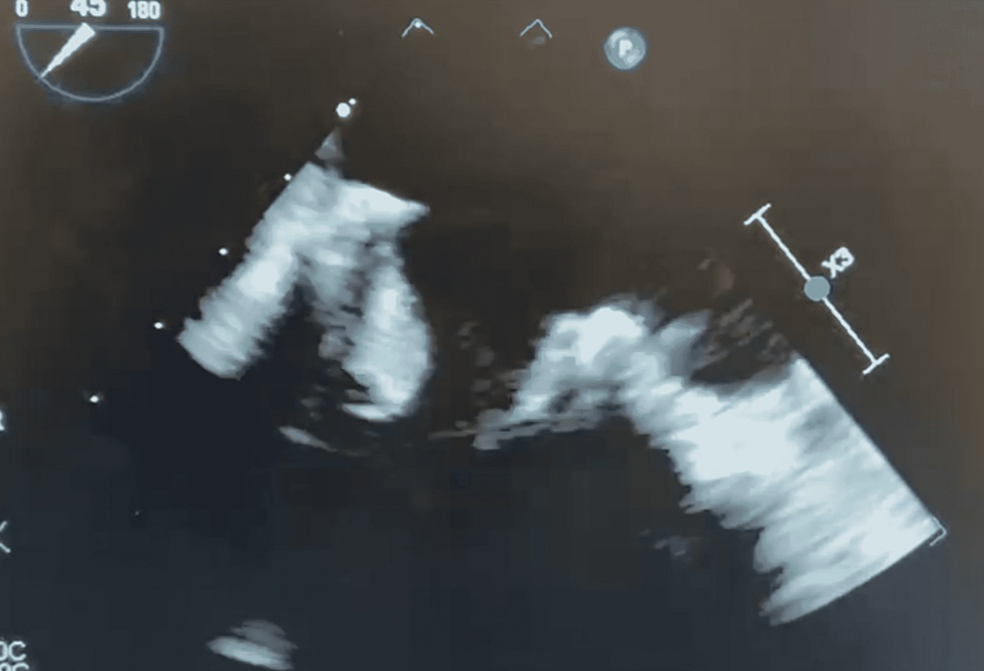
A transesophageal echocardiogram demonstrating a new 1.5 cm vegetation on the bioprosthetic mitral valve

 A transesophageal echocardiogram demonstrated a new 1.5 cm by 0.9cm vegetation on the anterior leaflet of the bioprosthetic mitral valve (Figure 2). Severe mitral stenosis with trace mitral regurgitation was also noted. 

Given the recent diagnosis of hemophagocytic lymphohistiocytosis, noninfectious etiologies were considered, such as marantic endocarditis or even an underlying malignancy or collagen vascular disease. However, further diagnostic workup was negative to indicate these conditions. Blood cultures resulted after 24 hours and again, showed no growth. A Karius test, which detects microbial cell-free DNA was completed to assess for a further range of microbial infections that cannot be detected by other measures. The results came back positive for the growth of the nontuberculous mycobacterium, *M. chimaera*. Accounting for the patient's history of recurrent vegetation, lung nodule, and systemic symptoms, his presentation was consistent with an *M.chimaera* infection. Although previous AFB blood cultures and prosthetic valve vegetations showed no growth, it is suspected that the patient had a systemic disseminated *M.chimaera* infection. 

The patient was then started on antimicrobial therapy with amikacin 800mg daily, azithromycin 500mg daily, ethambutol 1200mg daily, and rifabutin 300mg daily. The patient required valve replacement surgery; however, he was not considered a surgical candidate due to his multiple comorbidities. Unrelated, after the diagnosis of *M.chimaera* was made, the patient's lung biopsy came back significant for neuroendocrine cells. He was subsequently diagnosed with a neuroendocrine cell tumor and underwent radiation treatment for the tumor. The patient eventually made the decision to transition to hospice and ultimately expired. 

## Discussion

*Mycobacterium chimaera *is a gram-positive, acid-fast, classically nonmotile, slow-growing bacterium belonging to the* Mycobacterium* genus. It is typically described as a low virulence species. It is genetically similar to *Mycobacterium intracellulare* and is most often associated with pulmonary infections in the context of cystic fibrosis [4]. Healthcare-associated infections due to *M. chimaera* have recently come to the forefront in patients receiving cardiothoracic surgery with extracorporeal circulation and implantation of prosthetic materials. In 2015, a group of cases from Switzerland, Germany, the Netherlands, the UK, the USA, and Australia were linked to contamination of heating-cooling units produced at the LiveNova factory [5]. Since 2013, all cases have been linked to contamination of this specific brand (Stockert 3T, LivaNova, London, United Kingdom) of HCUs [6]. It was estimated that up to one-fourth of HCUs produced before 2014 were contaminated.

Manifestations of disseminated *M. chimaera* infection typically occur three months to five years after surgery and include endocarditis, wound or bone infection, splenomegaly, pancytopenia, hepatitis, renal involvement, and embolic phenomena [7-8]. Studies have shown that *M. chimaera* has a propensity to form robust biofilms, especially on implanted prosthetic devices. It has also been speculated that this mechanism contributes to its pathogenicity and resistance to standard antimycobacterial medications. Recent studies have also shown that *M. chimaera* can become motile and that this conversion contributes to the process of colonization and is important to reversible and irreversible cell adhesion. These mechanisms contribute to biofilm formation and bacterial survival within the environment [9].

Due to the lag time from surgery to symptom presentation, lack of effective diagnostic methods, and resistance to standard antimycobacterial therapies, the outcomes for patients with disseminated *M. chimaera* have been poor [5]. Scrivens et al. demonstrated that 18 out of 30 affected patients died within a median of 30 months after the initial surgery. Survival analysis identified younger age, mitral valve surgery, mechanical valve replacement, higher serum sodium concentration, and lower C-reactive protein as factors associated with better survival [1]. For the reasons listed above, patients undergoing open-chest cardiothoracic surgery that develop symptoms post-surgery should be evaluated for possible *M. chimaera* infection. In these cases, early therapy before systemic dissemination may improve outcomes, however, more data is needed.

## Conclusions

This patient was found to have disseminated *M. chimaera* infection six years after his initial mitral valve surgery. Unfortunately, without valve replacement, endocarditis cannot be cured. Chronic antimicrobial management is unlikely to eradicate prosthetic valvular disease. This patient was not considered a surgical candidate for a third valve surgery given the surgery itself would have a high mortality rate. This patient presented with the infection six years after his initial surgery. Therefore, *M. chimaera* should be excluded in patients who have undergone open-heart surgery who present with systemic symptoms such as fevers, even if the initial laboratory workup is negative for infectious etiologies.
